# Mucocutaneous leishmaniasis: case report and literature review of a rare endonasal infection

**DOI:** 10.11604/pamj.2020.36.292.24543

**Published:** 2020-08-17

**Authors:** Abrar Adnan Suqati, Annett Pudszuhn, Veit Maria Hofmann

**Affiliations:** 1 Department of Otorhinolaryngology, Head and Neck Surgery, Charité-Universitätsmedizin Berlin, Corporate Member of Freie Universität Berlin, Humboldt-Universität zu Berlin and Berlin Institute of Health, Campus Benjamin Franklin, Berlin, Germany

**Keywords:** Cutaneous infection, leishmaniasis, traveler’s diseases

## Abstract

Leishmaniasis is a protozoal infection transmitted by a sandfly vector. In Germany, leishmaniasis of the mucous membranes is a rare condition and usually due to extension of local skin disease into the mucosal tissue via direct extension, bloodstream or lymphatics. We report a case of endonasal leishmaniasis in a female German resident who presented in a university hospital with nasal obstruction. Histology of the left nasal septum biopsy was suggestive of leishmaniasis. The molecular detection of DNA was positive for leishmania infantum. The patient was successfully treated as a case of mucocutaneous leishmaniasis receiving liposomal amphotericin follow up visits showed significant improvement with no recurrence.

## Introduction

Leishmaniasis is a protozoal infection transmitted by a sandfly vector. According to the World Health Organization (WHO), mucocutaneous leishmaniasis (MCL) is endemic in Central and South America. Almost 90% of MCL cases occur in Bolivia, Brazil and Peru [1]. In Germany leishmaniasis of the mucous membranes is a rare condition and usually due to extension of local skin disease into the mucosal tissue via direct extension, bloodstream or lymphatics. Because of the chronic local destruction of facial structures, MCL may deface the patient if not recognized and adequately treated [2,3].

## Patient and observation

**Figure 1:**
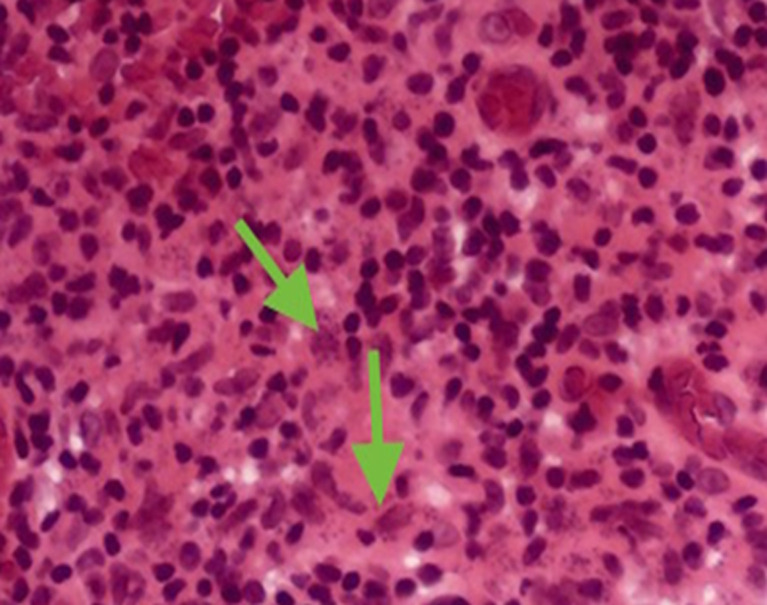
photomicrograph shows intracellular amastigote (hematoxylin-eosin stain)

A 66-year-old German woman presented in a university hospital with nasal obstruction. Clinically the patient showed nodular and ulcerative lesion at the nasal septum. Histology of the left nasal septum biopsy revealed necrosis, chronic inflammation and intracellular amastigote that was suggestive of leishmaniasis (Figure 1). The molecular-pathological detection of parasite DNA was positive for leishmania infantum. The medical history includes hypothyroidism and rheumatoid arthritis for which she was under continuous immunosuppressive treatment with methotrexate and anti-TNF-blocker (Humira®) several years ago. Moreover, she was treated for cutaneous leishmaniasis in the left elbow after a travel history in Mallorca five years ago (4 weeks, amphotericin B). The patient successfully retreated as a case of mucocutaneous leishmaniasis receiving at first liposomal amphotericin B for 2 weeks. Under the treatment showed the patient an increased level of creatinine, therefore, the therapy was changed to a phospholipid (Hexadecylphosphocholin = Miltefosin®) for 4 weeks with a side effect of vomiting. Follow up visits showed significant improvement with no recurrence.

## Discussion

Mucocutaneous leishmaniasis is rarely diagnosed in Germany. The majority of the cases are seen in patients living in or visiting endemic regions [3]. The reviewed medical literature through the electronic database Pubmed showed that leishmaniasis was not notifiable in Germany until 2000. Within 2 years 70 cases of leishmaniasis (43 cutaneous or mucocutaneous/mucosal, 27 visceral) were reported. The data were available for 58 patients (35 cutaneous or mucocutaneous/mucosal; 23 visceral). Harms *et al*. report of 35 patients with MCL or CL, 30 had a travel history to endemic areas and seventeen lesions were located in the face, including mouth and nose [4]. MCL developed as a consequence of cutaneous leishmaniasis. The literature review showed that the favorite sites for manifestations of this disease in the nose were the cartilaginous part of the nasal septum, the anterior portions of nasal fossa, the floor of the nose and the lower turbinate [5,6]. Patients with endonasal leishmaniasis complain nasal obstruction, epistaxis or rhinorrhea-impaired breathing as well as septal perforation. Misdiagnosis may lead to an unfavorable outcome [3,5,6]. Histologically, MCL may mimic other granulomatous or neoplastic diseases and differentiation is only possible by the morphological features of the parasite [6,7]. In majority of studies, the gold standard method of diagnosis was a biopsy with the molecular detection of parasite DNA by polymerase chain reaction (PCR) [2,8,9]. The primary goals of therapy for leishmaniasis is to prevent the mortality and morbidity. The first line of treatment for MCL is the pentavalent antimony compounds, liposomal amphotericin B or standard amphotericin B while miltefosine is used as a second line of treatment [9,10].

## Conclusion

MCL can lead to serious sequela, however, early diagnosis can prevent complications. The aim of this study to raise the awareness among the otolaryngologist in European countries of leishmaniasis of the mucous membranes as a differential diagnosis of mucocutaneous lesions especially in patients with travel history to endemic areas.
